# Seed Maturation Regulators Are Related to the Control of Seed Dormancy in Wheat (*Triticum aestivum* L.)

**DOI:** 10.1371/journal.pone.0107618

**Published:** 2014-09-11

**Authors:** Kazuhide Rikiishi, Masahiko Maekawa

**Affiliations:** Institute of Plant Science and Resources, Okayama University, Kurashiki, Okayama, Japan; Institute of Botany, Chinese Academy of Sciences, China

## Abstract

In Arabidopsis, the regulation network of the seed maturation program controls the induction of seed dormancy. Wheat EST sequences showing homology with the master regulators of seed maturation, *LEAFY COTYLEDON1* (*LEC1*), *LEC2* and *FUSCA3* (*FUS3*), were searched from databases and designated respectively as *TaL1L* (*LEC1-LIKE*), *TaL2L* (*LEC2-LIKE*), and *TaFUS3*. *TaL1LA*, *TaL2LA* and *TaFUS3* mainly expressed in seeds or embryos, with the expression limited to the early stages of seed development. Results show that tissue-specific and developmental-stage-dependent expressions are similar to those of seed maturation regulators in Arabidopsis. In wheat cultivars, the expression level of *TaL1LA* is correlated significantly with the germination index (GI) of whole seeds at 40 days after pollination (DAP) (*r* = –0.83**). Expression levels of *TaFUS3* and *TaL2LA* are significantly correlated respectively with GIs at 40 DAP and 50 DAP, except for dormant cultivars. No correlation was found between the expression level of *TaVP1*, orthologue of *ABA INSENSITIVE3* (*ABI3*), and seed dormancy. *DELAY OF GERMINATION1* (*DOG1*) was identified as a quantitative trait locus (QTL) for the regulation of seed dormancy in Arabidopsis. Its promoter has RY motif, which is a target sequence of *LEC2*. Significant correlation was found between the expression of *TaDOG1* and seed dormancy except for dormant cultivars. These results indicate that *TaL1LA*, *TaL2LA*, and *TaFUS3* are wheat orthologues of seed maturation regulators. The expressions of these genes affect the level of seed dormancy. Furthermore, the pathways, which involve seed maturation regulators and *TaDOG1*, are important for regulating seed dormancy in wheat.

## Introduction

Pre-harvest sprouting, the premature germination of seeds in the spike before harvest, is influenced by rainfall and temperature during seed development. Pre-harvest sprouting reduces yields and grain quality by increasing the activity of hydrolytic enzymes such as α-amylase, and produces a large economic loss [1]. Seed dormancy, which is an important factor in the control of pre-harvest sprouting, prevents germination under favorable conditions (e.g. moisture and temperature) [2]. It is an inherent capacity for adaptation to several environments. However, a high level of dormancy is economically undesirable because uniform and rapid germination are necessary for crop cultivation [3]. Therefore, modern cultivars have lower seed dormancy, which triggers pre-harvest sprouting. Sprouting damage is becoming a severe problem to the production of cereals worldwide. For these reasons, resistance to pre-harvest sprouting is a critical breeding objective in the cultivation of many cereals including wheat and barley.

A plant hormone, abscisic acid (ABA), plays a fundamental role in the induction and maintenance of dormancy. In Arabidopsis, many genes involved in the biosynthesis (*ABA1*, *ABA2*, *NCEDs*), catabolism (*CYP707A*), and signaling (*ABIs*, *ERA*, *ABH*) have been identified [4]–[10]. Recent molecular genetic approaches suggest that the pathway independent of plant hormones as well as plant hormone dependent pathways are important for the control of seed dormancy [11]. Dormancy, which is induced early during seed maturation, increases until the seed is fully developed [12]. Seed dormancy is affected by the seed maturation program, which is controlled by at least four major regulators in Arabidopsis [13], [14], [15]. *LEAFY COTYLEDON2* (*LEC2*), *FUSCA3* (*FUS3*) and *ABA INSENSITIVE3* (*ABI3*) are plant-specific transcription factors with B3 domain [16]–[18]. *LEC1* encodes the HAP3 subunit of the CCAAT-binding transcription factor [19]. Mutations of seed maturation regulators have pleiotropic effects, and the mutants show several morphological and physiological defects. Actually, *lec1*, *lec2* and *fus3* mutants represent defects of cotyledon identity, loss of desiccation tolerance, and reduction of seed dormancy [18]–[27]. Furthermore, *abi3* mutants show the reduction of desiccation tolerance and seed dormancy. In addition, ABA insensitivity and defects of chlorophyll degradation with normal cotyledon morphology were observed in *abi3* mutants.

Transcription factors with B3 domain reveal distinct temporal expression patterns during seed development. Expression of *ABI3* is maintained at a higher level through seed maturation, but expression of *FUS3* is reduced before maturation [13], [17], [28]. *LEC2* shows the peak of expression before the highest expression of *FUS3* and *ABI3*. Furthermore, *ABI3* and *FUS3* preferentially express, respectively, in the whole embryo and protoderm of embryo [15], [28], [29]. *LEC2* is expressed mainly during early embryo development [13], [18]. Therefore, B3 domain family genes have different traits for temporal expression.

*LEC2* and *FUS3* affect the accumulation of several hormones by regulating distinct pathways. *FUS3* negatively regulates the accumulation of active form gibberellin (GA) by suppressing *GA3ox2*, and it positively regulates the accumulation of ABA [26], [30], [31]. *LEC2* also suppresses the expression of *GA3ox2* and acts as a positive regulator of auxin biosynthesis [30], [32]. *LEC1* regulates the expressions of *ABI3* and *FUS3*, and its peak expression is observed before up-regulation of B3 domain factors [13], [33]. These expression patterns suggest that *LEC1* acts on the upstream of B3 regulation network. However, overexpression of *LEC2* ectopically activates the embryonic pathways that are regulated by *LEC1* [32]. Intricate networks are constructed by seed maturation regulators.

The B3 domain recognizes consensus RY/Sph sequence implicated in the seed specific expression. RY motif, which is involved in the promoter of maturation RNAs expressed during the maturation phase of seed development, is regulated directly by *LEC2* [34]. *DELAY OF GERMINATION1* (*DOG1*) has been identified as a quantitative trait locus (QTL) controlling the natural variation of seed dormancy in Arabidopsis [35]. DOG1 protein abundance in freshly harvested seeds acts as a timer for seed dormancy release [36]. In fact, *DOG1* also has RY motif in its promoter similar to the seed maturation RNAs. The expression of *DOG1* is controlled by B3 domain factors [35]. The regulation pathways involve B3 transcription factors; *DOG1* plays an important role in connecting the control of seed maturation and dormancy.

Rikiishi *et al*. [37] demonstrated that the amount of transcripts of a seed specific transcription factor, *TaABF* (*ABRE Binding Factor*) *1*, is related to seed dormancy levels in wheat seeds. Several seed-specific transcription factors show fluctuating expressions depending on the developmental stage. Such expressions are determined by the maturation program, but seed maturation regulators, except for *ABI3*/*VIVIPAROUS1* (*VP1*), have not been investigated in wheat. The present study investigated the expressions of master regulators of seed maturation, *LEC1*, *LEC2*, *FUS3* and *ABI3*/*VP1*, in relation to wheat seed dormancy. EST sequences showing homology with Arabidopsis seed maturation regulators were searched from databases. *TaL1L* (*LEC1-LIKE*), *TaL2L* (*LEC2-LIKE*), and *TaFUS3* were expressed mainly in seeds or embryos. Their expression was limited to the early stages of seed development, suggesting that these ESTs are orthologues of seed maturation regulators in wheat. For monocot species, a few studies have examined seed maturation regulators such as *ZmLEC1* in maize [38], *HvFUS3* in barley [39], and *OsLFL1* in rice [40], [41]. *LEC2* orthologue has not been identified in monocots. At present, *TaL2LA* is the first orthologue of *LEC2* identified in a monocot species. Expression levels of wheat seed maturation regulators were examined in wheat cultivars showing different levels of seed dormancy. Then the potential effects of these expressions on dormancy were discussed.

## Materials and Methods

### Plant materials

Wheat cultivars, AUS1408 (AUS), Chihokukomugi (Chi), Chinese Spring (CS), Kitakei-1354 (Kit), Norin61 (N61), OW104 (OW), RL4137 (RL), Tamaizumi (Tam), and Zenkoujikomugi (Zen) were grown in a field under a plastic roof to protect them from rainfall. Seeds were sown in plastic trays in mid-November. Then 20 seedlings of each cultivar were transplanted to the field in mid-December. Spikes were tagged at anthesis. Seeds were harvested every 5 days from 35 days after pollination (DAP) to 50 DAP and then dried in an oven at 150°C for 3 hr to determine the water contents. To ensure the uniformity of developmental stage, seeds were collected only from primary and secondary florets of the center spikelets.

### Germination test

Ten whole seeds and ten half seeds that had been cut transversely and contained the embryos were placed on filter paper in a Petri dish containing 6 ml of distilled water. The Petri dishes were incubated in the dark at 20°C. All germination tests had three replications. Germinated seeds were counted daily for 14 days. A weighted germination index (GI) was calculated to assign maximum weight to seeds that had germinated first and to assign less weight to those that germinated subsequently, as described by Rikiishi and Maekawa [42]. The GI values were converted into arcsine-transformed values and were used for statistical analyses.

### RNA isolation and real-time quantitative RT-PCR analysis

Total RNA was isolated from 5 seeds at 10 DAP and 20 embryos at 20–50 DAP, and from 0.2 g of leaf and root tissues derived from Norin61 seedlings grown in the dark for 10 days using commercial kit (FastRNA Pro Green; Qbiogene Inc.). The mRNA was purified from 20 µg of total RNA (Poly (A) Purist MAG; Ambion Inc.). First-strand cDNA was prepared using a reagent kit (PrimeScript RT; Takara Bio Inc.). For quantitative RT-PCR, 5 µL of diluted cDNA (1∶25), 0.2 µM of each primer, and 10 µL of SYBER PremixExTaq (Takara Bio Inc.) with a total reaction volume of 20 µL was used in triplicate. Subsequently, PCR was performed (Light Cycler 2.0; Roche Diagnostics Corp.). The condition of the PCR reaction was 95°C for 10 s, followed by 40 cycles of 95°C for 5 s and 60°C for 20 s. Gene-specific primers designed with Primer Express software (Applied Biosystems) are listed in Table S1. The primers of *TaVP1* were designed in the consensus regions among sequences derived from A, B and D genomes. The relative amount of each target transcript was determined by generating standard curves using a dilution series of amplified products of the target sequence. The amount of 10^10^ times dilution was defined as 1.0. Quantifications were performed using three biological replications. All quantifications were normalized to the amplification of *TaCDCP (Cell Division Control Protein)* [43]. The accession numbers of *TaCDCP* and *TaDOG1* are, respectively, Ta54227 (Unigene Cluster) and RFL_Contig1498 (TriFLBD). All kits were used according to the respective manufacturer’s protocol.

### Sequence analysis

Sequences of ESTs were obtained from searches of NCBI/GenBank/Blast/PlantGDB/TriFLDB databases. Accession numbers of *LEC1*, *LEC2*, *FUS3*, *ABI3*, *VP1*, and the respective EST sequences are listed in Table S2. Deduced amino acid sequences of the ESTs were compared with that of corresponding Arabidopsis gene using the bl2seq BLAST program (http://blast.ncbi.nlm.gov/) and similarity was evaluated by Query coverage (%) and E-value. Alignments of B and B3 domains in the deduced amino acid sequences were performed using GENETYX ver. 9 software (Genetyx Corp., Tokyo). Based on aligned sequences, an unrooted phylogenetic tree was constructed using Neighbor-Joining (NJ) method with 100 replications.

## Results

### 1. Search for wheat orthologues of seed maturation regulators

**Figure 1:**
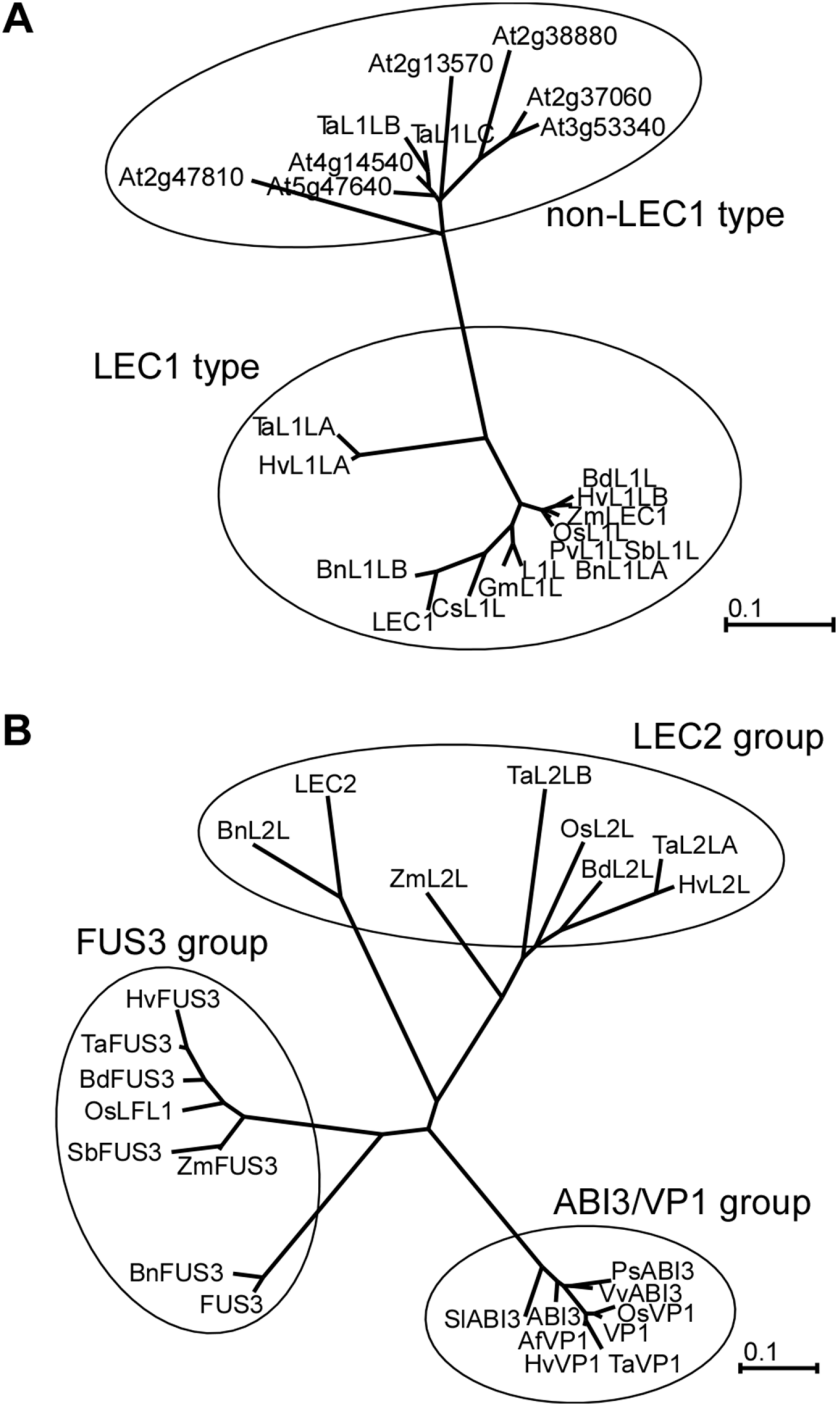
Unrooted phylogenetic trees based on the sequences of conserved domains. Trees were constructed from the deduced amino acid sequences of the B domains of LEC1 type, non-LEC1 type and their orthologues (A) and of the B3 domains of Arabidopsis *AFL* (*ABI3*/*FUS3*/*LEC2*) group genes and their orthologues (B). Phylogenetic trees were generated by Neighbor-Joining method. Scale bars represent amino acid substitutions per site. Abbreviations represent species names as follows: Af, *Avena fatua*; Bd, *Brachypodium distachyon*; Bn, *Brassica napus*; Cs, *Cucumis sativus*; Gm, *Glycine max*; Hv, *Hordeum vulgare*; Os, *Oryza sativa*; Ps, *Pisum sativum*; Pv, *Panicum virgatum*; Sb, *Sorghum bicolor*; Sl, *Solanum lycopersicum*; Ta, *Triticum aestivum*; Vv, *Vitis vinifera*; Zm, *Zea mays*.

EST sequences showing higher homology with seed maturation regulators of Arabidopsis were sought from several databases. In Arabidopsis, *LEC1* and its homologous genes form B domain family. The B domain family genes were categorized into LEC1 type and non-LEC1 type. The *LEC1-LIKE* (*L1L*) had been identified as homologous gene of *LEC1,* thus *L1L* was categorized into LEC1 type; 83.3% of the amino acid sequences in B domain of *L1L* are identical with that of *LEC1*. Fourteen EST sequences representing higher homology with the B domain of *LEC1* were found from 10 species (Fig. S1) and are designated as *L1L*. Deduced amino acid sequences showed less homology with *LEC1* in the region outside of the B domain. In the sequence of B domain, *ZmLEC1*, a maize orthologue, revealed 83.3% amino acid sequences identity with *LEC1*. Amino acid sequences of wheat ESTs, *TaL1LA*/*B*/*C*, were 74.4%, 62.2% and 65.6%, respectively, identical with *LEC1* in the B domain sequence. The percentages of homology are lower than that of *ZmLEC1*. The amino acid sequence of *TaL1LA* and *L1L* showed 74.4% identity in the B domain. *TaL1LA* showed similar level of homology with both *LEC1* and *L1L*. The B domains of *TaL1LB* and *TaL1LC* showed higher homology with *L1L* (71.1% and 70.0%, respectively) than that with *LEC1*. A phylogenetic tree was constructed for B domain sequences of the ESTs together with *LEC1*, *L1L*, and 7 non-LEC1 type genes of Arabidopsis (Fig. 1A). *TaL1LA*, *HvL1L*, and *ZmLEC1* were classified as LEC1 type, whereas *TaL1LB* and *TaL1LC* belonged to the non-LEC1 type.

*HvFUS3* had been identified as a barley orthologue of *FUS3*. The EST with accession number JV941539 was found to have higher homology with *HvFUS3*. Deduced amino acid sequence of the EST showed 91.5% identity with *HvFUS3*. Therefore, the EST was designated as *TaFUS3*. Amino acid sequences of *TaFUS3* and *HvFUS3* in the B domain showed 61.2% and 63.1% identity with that of *FUS3* of Arabidopsis, respectively.

Seven EST sequences that are found to have homology with *LEC2* (Fig. S2) were designated as *L2L* (*LEC2-LIKE*). The EST with accession number of HM370539 of *Brassica napus* (*BnL2L*) showed highest homology with *LEC2*, and 66.5% and 77.7% of the deduced amino acid sequences were found to be identical with CDS and B3 domain sequences, respectively. In other *L2Ls*, the sequences outside of the B3 domain showed lower homology with *LEC2*. The higher homology was detected only in the B3 domain. The B3 domain sequences of *L2Ls* showed 47.6% (*TaL2LA*, *TaL2LB*) to 53.4% (*BdL2L*) identity with *LEC2*. In fact, B3 domains of *L2Ls* showed 44.7–52.4% and 52.4–58.3% identity with those of *FUS3* and *ABI3*, respectively. The B3 domain sequences of *L2Ls* showed similar homology with those of *LEC2*, *FUS3*, and *ABI3*. Therefore, B3 domain sequences of *LEC2*, *FUS3*, *ABI3*/*VP1* and their homologous EST sequences were classified into three groups using phylogenetic analysis (Fig. 1B). Our results showed that *TaL2LA* and *TaL2LB* belonged to the same group as *LEC2*. Sequence analyses results indicated that *TaL1LA*, *TaL2LA*/*B* and *TaFUS3* are respective candidates of *LEC1*, *LEC2*, and *FUS3* orthologues.

### 2. Expression analysis of seed maturation regulators

**Figure 2:**
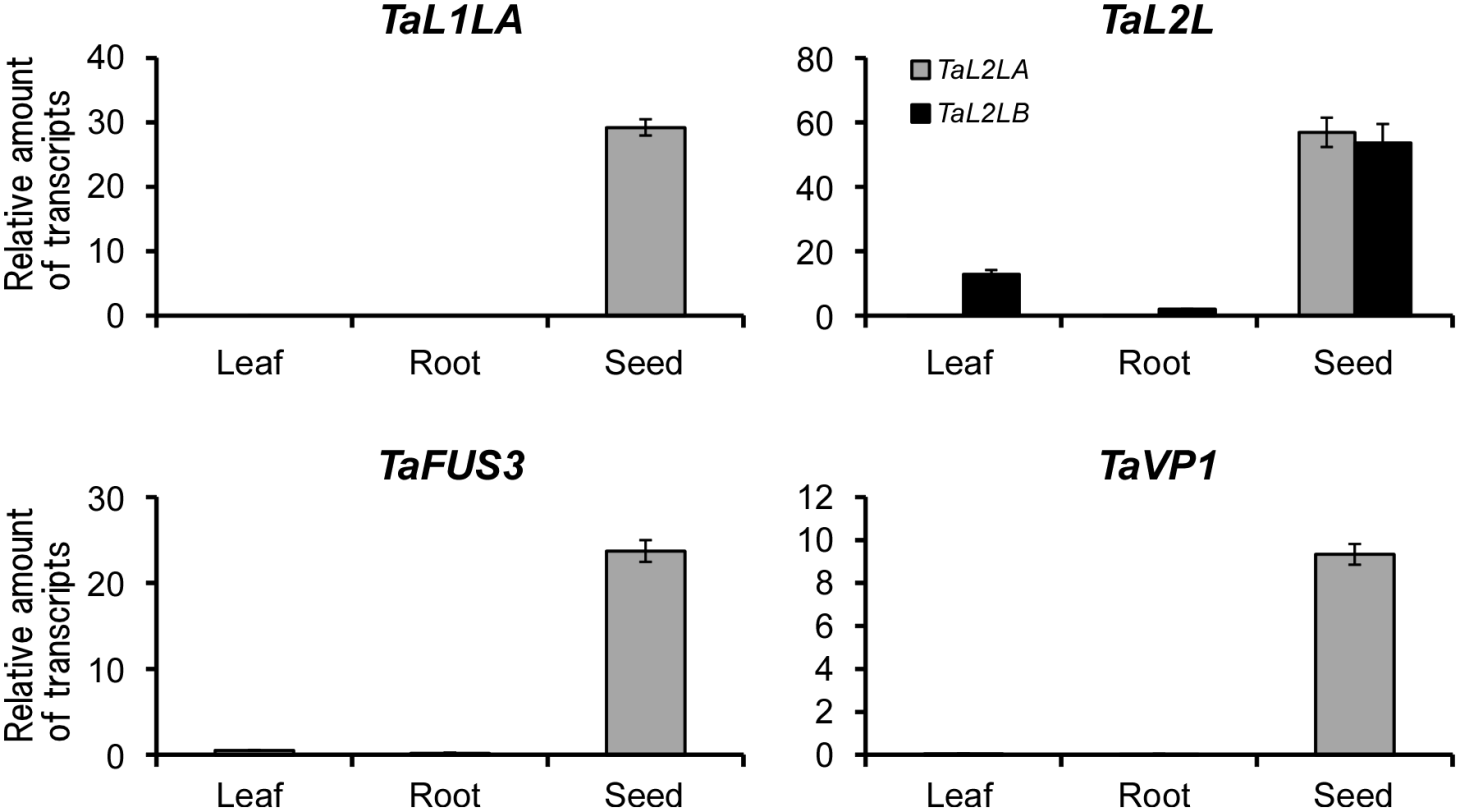
Relative expressions of wheat orthologues of seed maturation regulators in different tissues. Relative amounts of transcripts were determined in leaves, roots, whole seeds at 10 DAP (*TaL1LA*, *TaL2LA*) and embryos at 20 DAP (*TaL2LB*, *TaFUS3*, *TaVP1*). Bars represent SE.

**Figure 3:**
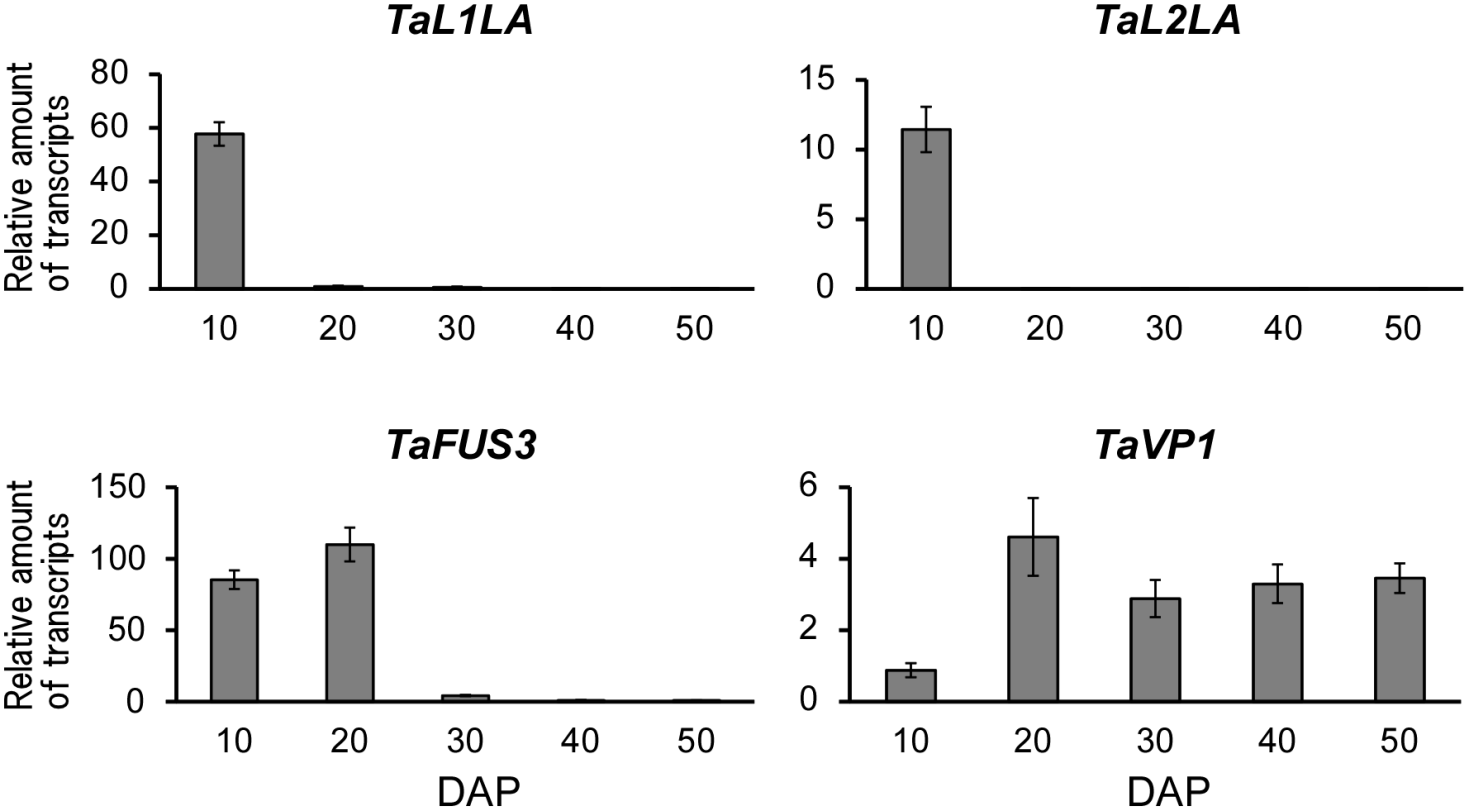
Relative expressions of wheat orthologues of seed maturation regulators at different seed developmental stages. Relative amounts of transcripts were determined in whole seeds at 10 DAP and in embryos at 20–50 DAP. Bars represent SE.

Expression levels of candidate genes were determined in several tissues. *TaL1LA*, *TaL2LA* and *TaVP1* were expressed in seeds (10 DAP) or embryos (20–50 DAP) and showed no expression in leaf or root tissues (Fig. 2). Although *TaFUS3* also showed higher expression in seeds and embryos, faint expression of this gene was detected in leaf and root tissues. However, *TaL2LB* was expressed in seed, leaf, and root tissues. For temporal expression patterns during seed development, *TaL1LA* and *TaL2LA* showed expression at 10 DAP. The expressions were decreased rapidly at 20 DAP (Fig. 3). Expression of *TaFUS3* started at 10 DAP. The highest expression was observed at 20 DAP. *TaFUS3* was expressed at low level at 30 DAP and thereafter. Although *TaVP1* was expressed at a lower level at 10 DAP, a higher level in the remaining seed developmental stages (20–50 DAP) was observed. *TaL2LB* was expressed at all developmental stages (data not shown). The expression of *TaL2LB* showed different patterns from that of *TaL2LA* in terms of developmental stage and tissue specific expressions.

### 3. Relation between the expression of seed maturation regulators and seed dormancy in wheat cultivars

**Figure 4:**
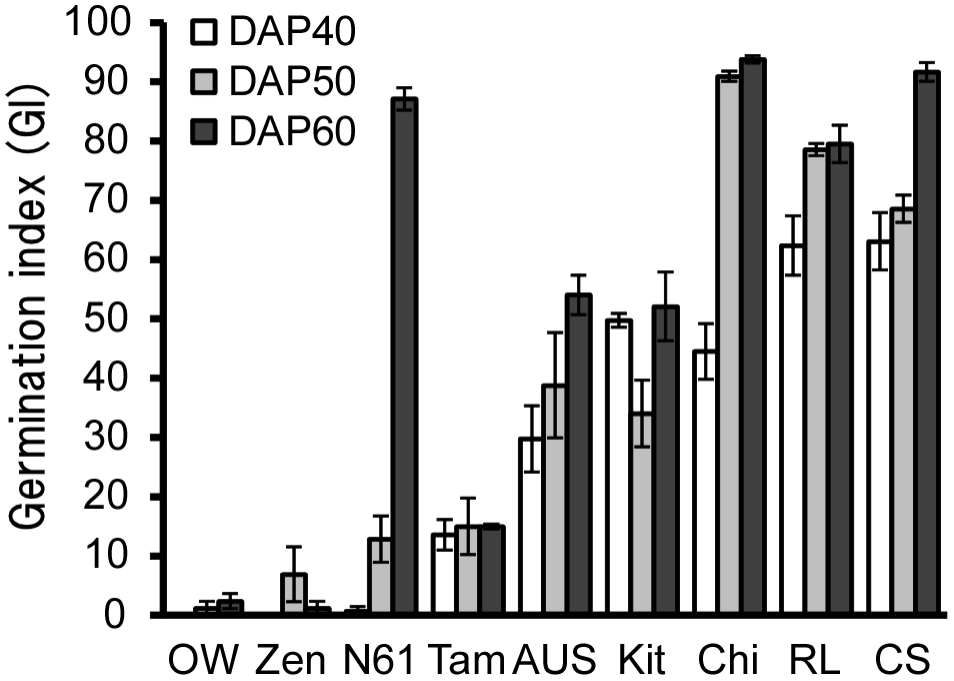
Germination index of whole seeds at 40–60 DAP in wheat cultivars. AUS, AUS1408; Chi, Chihokukomugi; CS, Chinese Spring; Kit, Kitakei-1354; N61, Norin61; OW, OW104; RL, RL4137; Tam, Tamaizumi; Zen, Zenkoujikomugi. Bars represent SE.

**Figure 5:**
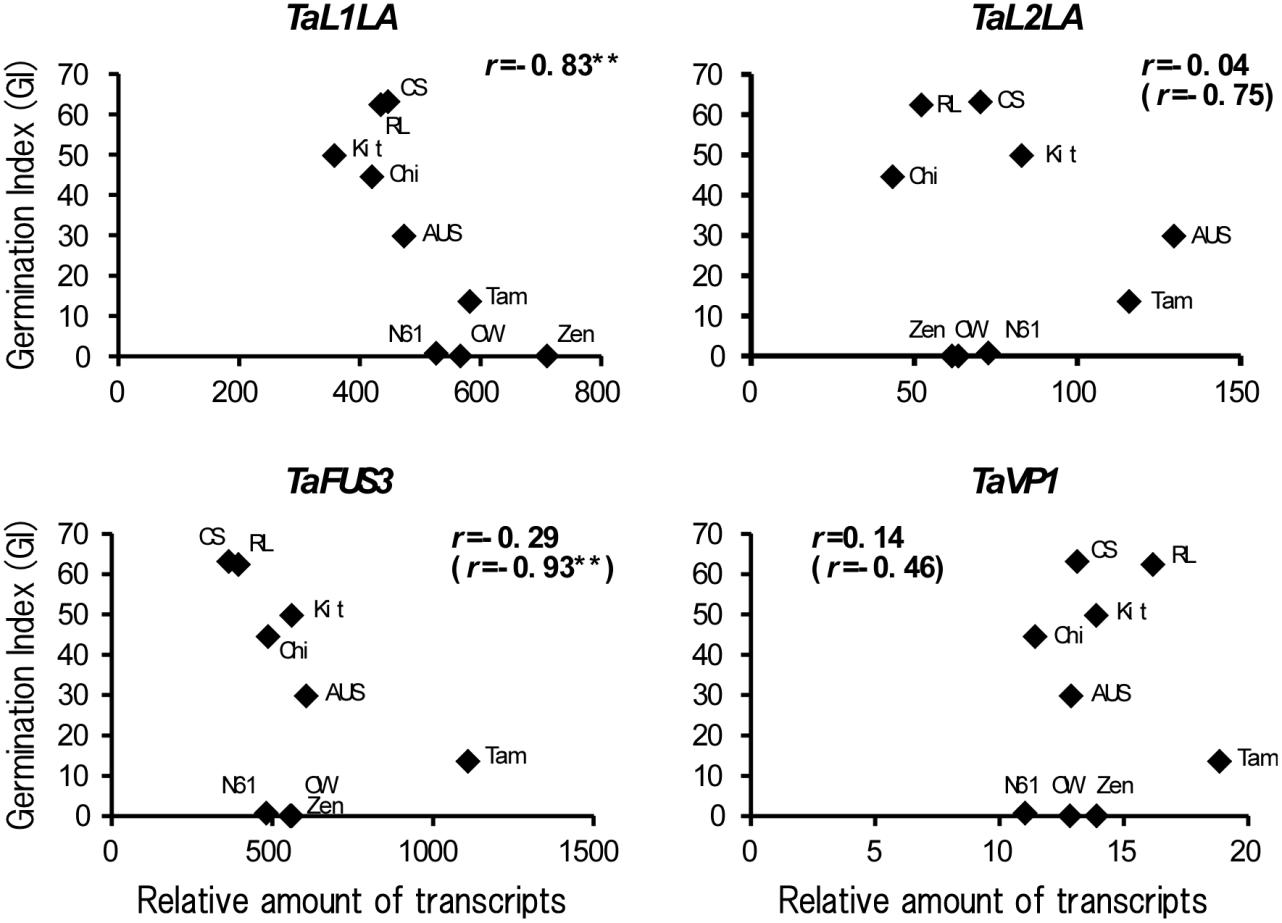
Relation between dormancy and relative expression of wheat orthologues of seed maturation regulators. Dormancy represents germination index (GI) of whole seeds at 40 DAP. Numerals in parentheses represent correlation coefficients, except for dormant cultivars, N61, OW, and Zen. Asterisks (**) denote significance at the 1% level.

**Figure 6:**
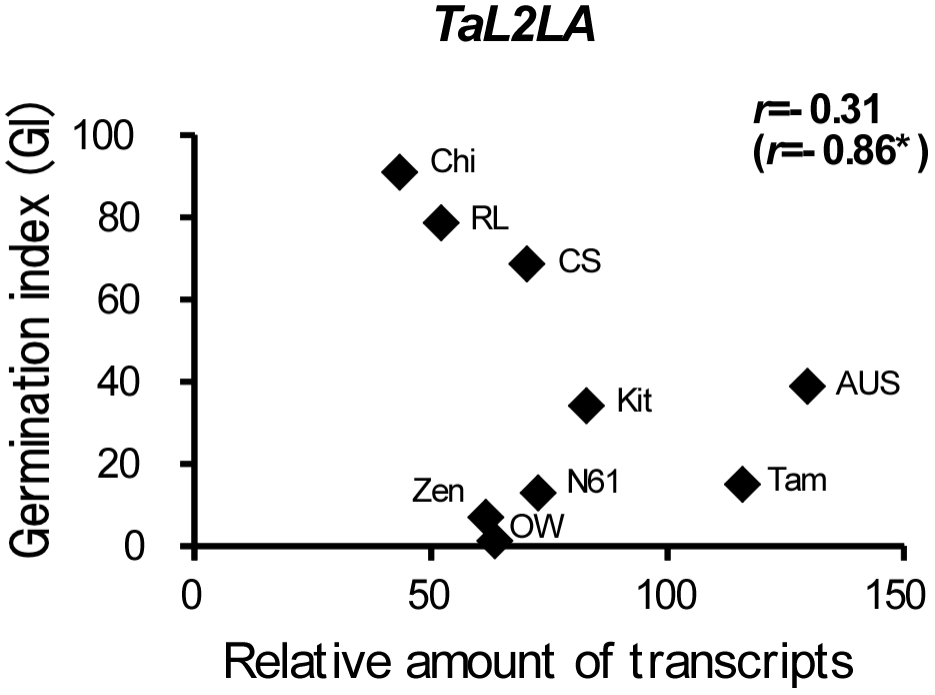
Relation between dormancy and relative expression of *TaL2LA*. Dormancy represents germination index (GI) of whole seeds at 50 DAP. Numeral in parentheses represent correlation coefficients, except for dormant cultivars, N61, OW, and Zen. Asterisks (*) denote significance at the 5% level.

Water contents of developing seeds were determined in nine wheat cultivars. All cultivars showed less than 20% at 40–45 DAP. Germination potential was evaluated by the germination index (GI) of half seeds, which were released from dormancy. Germination was observed at 20 DAP and the later stages in all cultivars. GIs of half seeds were low (20.2–37.0) at 20–30 DAP, but increased at 40 DAP (71.3–90.2). GIs of whole seeds (40–60 DAP) from the cultivars that exhibited different levels of seed dormancy varied (Fig. 4). Higher GIs were observed in Chi (44.5–93.8), RL (62.4–79.5), and CS (63.1–91.7), indicating that the levels of seed dormancy in these cultivars were low. Whereas, OW (0–2.4), Zen (0–6.9), and Tam (13.6–15.0) showed lower GIs and retained strong dormancy at 40–60 DAP. Although N61 also showed low GIs (0.7 and 12.9) at 40–50 DAP, the GI increased at 60 DAP (87.1). Dormancy was rapidly released at 60 DAP in N61. Seeds of AUS and Kit showed intermediate GIs and moderate dormancy levels. Relations between dormancy and expression levels of seed maturation regulators were examined in cultivars (Fig. 5). Expression of *TaL1LA* showed significant correlation with GIs at 40 DAP (*r* = –0.83**). Higher expressions tended to be associated with a higher level of seed dormancy. No significant correlation was observed in the cases of *TaL2LA*, *TaFUS3*, or *TaVP1*. However, the expressions of *TaFUS3* were correlated significantly with GIs except for dormant cultivars, N61, OW, and Zen (*r* = –0.93**). Expression of *TaL1LA* also showed a significant correlation with GIs at 50 DAP (*r* = –0.70*). Although no significant correlation was observed between expressions of *TaFUS3* and *TaVP1* and GIs at 50 DAP, the expression of *TaL2LA* was correlated significantly with GIs (*r* = –0.86*), except for dormant cultivars as observed for *TaFUS3* (Fig. 6).

### 4. Expression analysis of *DOG1* orthologue

**Figure 7:**
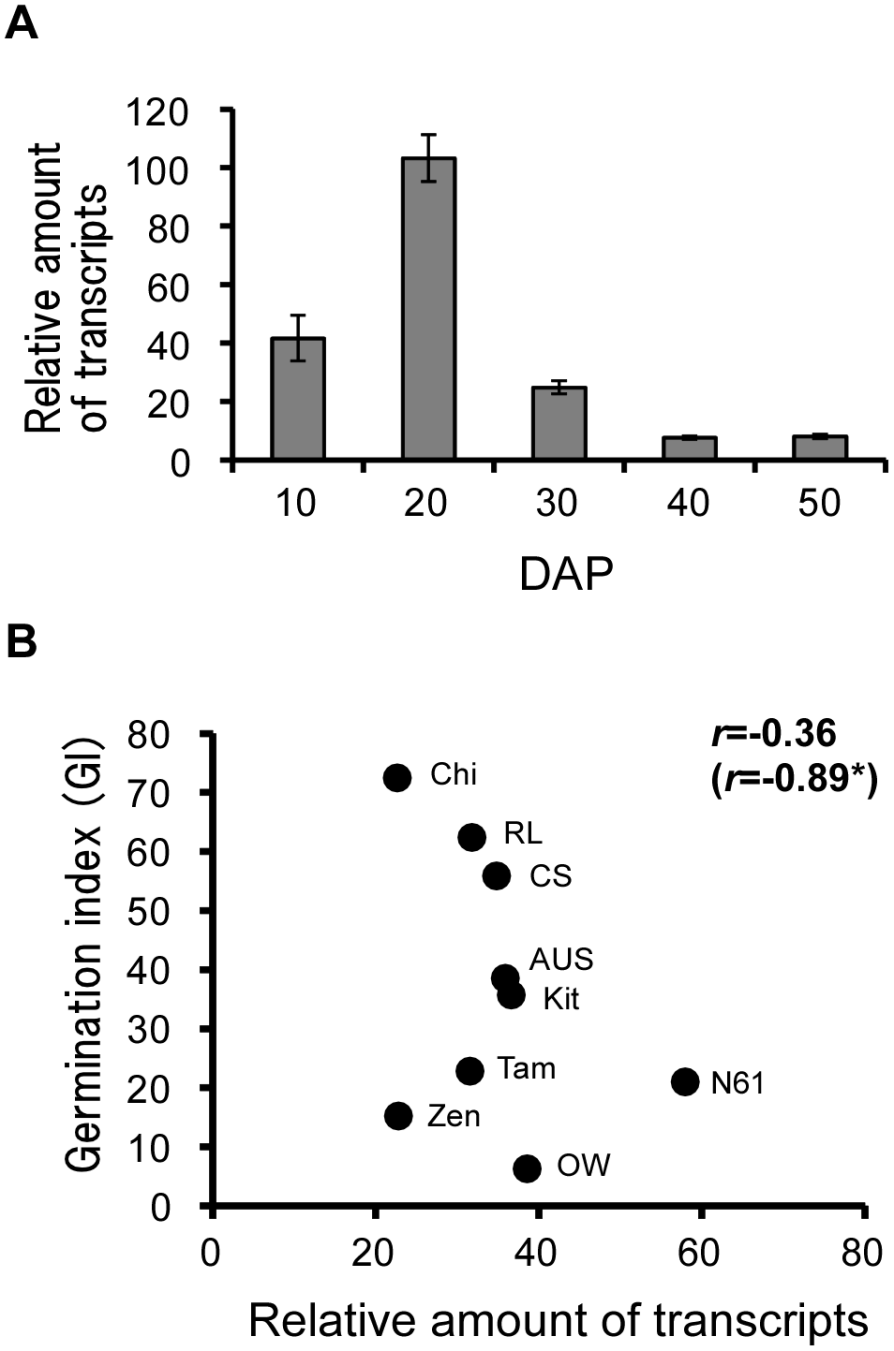
Expression analysis of *TaDOG1*. A: Relative expressions of *TaDOG1* at different stages of seed development. Bars represent SE. B: Relation between relative expressions of transcripts and germination index (GI) of whole seeds at 50 DAP in wheat cultivars. Numeral in parentheses represent correlation coefficients, except for dormant cultivars, OW, Tam, and Zen. Asterisks (*) denote a significance at the 5% level.

*TaDOG1* was expressed mainly in the embryos (Rikiishi and Maekawa, 2010). Our results showed peak expression of *TaDOG1* at 20 DAP. The expression was decreased during the later developmental stages (Fig. 7A). The highest expression levels of *TaDOG1* showed no significant correlation with GIs of whole seeds. However, significant correlation was detected between its expression levels and GIs of whole seeds at 50 DAP in six cultivars except for dormant cultivars, OW, Tam, and Zen (Fig. 7B).

## Discussion

Seed maturation is regulated by at least four master regulators in Arabidopsis. These master regulators are classified into two groups based on the conserved domain. *LEC1* encodes HAP3 subunit of CCAAT-binding factor with B domain. Other regulators, *LEC2*, *FUS3*, and *ABI3* belong in the group of transcription factor with B3 domain. These regulators control the seed maturation, dormancy, and desiccation tolerance [12], [13], [15], [22]–[24]. In the present study, wheat orthologues of seed maturation regulators were identified. Relations between their expressions and seed dormancy were examined.

The B domain of *LEC1* is conserved in eukaryotes [19]. Genes encoding HAP3 subunit were classified into LEC1 type (*LEC1* and *L1L*) and non-LEC1 type in Arabidopsis [33]. *L1L* was able to rescue the mutant phenotypes of *lec1*. However, non-LEC1 type genes did not complement *LEC1* functions. *L1L* has similar functions as *LEC1*. The functions of non-LEC1 type are distinct from that of *LEC1*. *TaL1LA*, *TaL1LB*, and *TaL1LC* are homologous EST sequences with *LEC1*. *TaL1LA* and *TaL1LB*/*C* are classified, respectively, as LEC1 type and non-LEC1 type. Furthermore, *TaL1LA* is expressed specifically in seeds, in which the expression is limited to 10 DAP during seed development. In Arabidopsis, *LEC1* also showed seed-specific expression. Transcripts were detected early during embryogenesis [19], [44]. However, *L1L* is expressed mainly in the seeds, but weak expression was also observed in the other tissues [33]. Tissue and temporal specific expressions indicated that *TaL1LA* is a wheat orthologue of *LEC1*.

*HvFUS3* had been identified as barley orthologue of *FUS3*, and the functions of *HvFUS3* are similar to that of *FUS3* [39]. *TaFUS3* showed higher homology with *HvFUS3* and showed seed specific expression. The expression of *TaFUS3* was detected at the early stage of seed development. *TaVP1* had been identified by McKibbin *et al*. [45]. Expression of *TaVP1* was maintained in higher levels throughout the maturation phase of seed development, similar to that of *ABI3* [28]. No orthologue of *LEC2* has been identified in monocot species. In the present study, *TaL2LA* and *TaL2LB* are found to be wheat ESTs showing homology with the B3 domain of *LEC2*. Although the B3 domain sequences of *TaL2LA* and *TaL2LB* showed similar levels of homology with *LEC2*, *FUS3*, and *ABI3*; *TaL2Ls* are classified by our phylogenetic analysis into the same group of *LEC2*. *LEC2* showed the highest expression at 8–9 DAP, which showed a decline at the later developmental stages. No transcript of *LEC2* was detected in the dry seeds of Arabidopsis [13], [18]. *TaL2LA* was specifically expressed in the seeds. However, the expression was limited to the early stage of seed development. These results indicate that *TaL2LA* is a wheat orthologue of *LEC2*. Kroj *et al*. [13] reported that the expression of *LEC2* precedes that of *FUS3* and *ABI3* during embryo development. Among our results, *TaFUS3* showed the highest expression at 20 DAP, although the peak expression of *TaL2LA* was observed at 10 DAP. Sequential expressions of *TaL2LA* and *TaFUS3* were similar to those of *LEC2* and *FUS3*, supporting that these genes are orthologues of seed maturation regulators. *TaL2LB* showed different expression patterns from that of *TaL2LA* and *LEC2*. Plant specific transcription factors with B3 domain include several gene families such as *AFL* (*ABI3*/*FUS3*/*LEC2*), *VAL* (*VP1*/*ABI3-Like*), *RAV* (*Related to ABI3*/*VP1*), *ARF* (*Auxin Response Factor*), and *REM* (*Reproductive Meristem*) [46]. These transcription factors control several growth and development aspects in Arabidopsis. Diverse functions of B3 domain genes suggest that *TaL2LB* has other functions than regulating seed dormancy.

Expression levels of seed maturation regulators are compared with seed dormancy levels in wheat cultivars. Expression levels of *TaL1LA* showed significant correlation with GI of whole seeds. Expression levels of *TaL2LA* and *TaFUS3* were also correlated significantly with seed dormancy levels, except for dormant cultivars. Taking into account these facts and our observations together, the expression levels of *TaL1LA*, *TaL2LA*, and *TaFUS3* affect the levels of seed dormancy in wheat cultivars. N61, Zen, and OW showed strong dormancy; nevertheless, the expression levels of *TaL2LA* and *TaFUS3* in these cultivars were similar to those of moderate cultivars. Baumbusch *et al*. [47] reported that the expressions of *LEC1*, *FUS3*, and *ABI3* do not differ among ecotypes (Ws, C24, Cvi), that show large variation in dormancy levels. The after-ripening requirement of Cvi was distinct from those of other accessions in Arabidopsis [48], suggesting that the level of dormancy might be determined by different regulatory pathways among ecotypes. Some of those pathways might be divergent from those for seed maturation regulators. Dormancy levels of OW and Zen cultivars were found to be distinct from other cultivars because poor germination was observed at 60 DAP. In these cultivars, dormancy levels might be regulated mainly by a pathway that is independent from that of seed maturation regulators.

Jones *et al*. [49] demonstrated that the mRNA level of *AfVP1* has strong correlation with seed dormancy level in wild oat, *Avena fatua*. However, relative amounts of *TaVP1* transcripts were unrelated to seed dormancy in our experiments. Liu *et al*. [50] also reported that the expression of *TaVP1* is not related to dormancy release by after-ripening. McKibbin *et al*. [45] reported that alternative splicing of *TaVP1* may contribute to increased susceptibility to pre-harvest sprouting. In wheat, the abundance of misspliced transcripts might affect the function of *TaVP1*.

Studies of the regulation networks of seed maturation have indicated that *LEC1*, which acts as a primary regulator of *FUS3* and *ABI3*, regulates early embryogenesis, desiccation tolerance and accumulation of seed storage proteins. However, *LEC2* is not involved in these pathways [51]. *LEC2* regulates triacylglycerol and fatty acid biosynthesis, as well as the synthesis of seed storage proteins independent of the regulation pathway of *LEC1*. Our results show that, although the expression levels of *TaL1LA* and *TaFUS3* are significantly correlated with GIs at 40 DAP, *TaL2LA* showed correlation with GIs at 50 DAP. The expressions of *TaL1LA*/*TaFUS3* and *TaL2LA* affect the levels of seed dormancy at different developmental stages. Thus, *TaL1LA* and *TaL2LA* might act on the different regulatory pathways similar to those of Arabidopsis. However, the regulatory networks of seed maturation remain unknown in monocot species. In fact, only few studies have examined seed maturation regulators in monocots, including *ZmLEC1* [38], *HvFUS3* [39], and *OsLFL1* [40], [41], apart from the orthologues of *ABI3*/*VP1*. Furthermore, no orthologue of *LEC2* has been identified in monocot species [51]. *TaL2LA* is the first orthologue of *LEC2* in monocot. No report has described the association of the expression of four seed maturation regulators with seed dormancy in monocots. Regulatory networks of seed maturation can be compared between monocots and dicots in further investigations.

*DOG1* has been identified as a QTL for the regulation of seed dormancy. In fact, *DOG1* expression is associated with the geographical variation of seed dormancy in Arabidopsis accessions [35], [52]. Rikiishi and Maekawa [42] reported that *TaDOG1* is related to the regulation of seed dormancy in wheat. *DOG1* has a RY motif in the promoter. RY motif is a target sequence of B3 domain type transcription factors, suggesting that *DOG1* is under the control of *LEC2* in Arabidopsis [13], [34], [35], [53]. In the present study, the expression of *TaDOG1* showed a significant correlation with dormancy level except for dormant cultivars. Such correlation is similar to that observed with seed maturation regulators. Furthermore, Rikiishi *et al*. [37] demonstrated that the expression of bZIP type transcription factor, *TaABF1* is related to the level of seed dormancy in wheat. *LEC1* conferred ABA inducibility to target genes by recruiting ABRE binding factor such as bZIP [54], [55]. *TaDOG1* and *TaABF1* might act on the regulation of seed dormancy under the control of seed maturation regulators. The regulatory networks of seed maturation might be conserved for the control of seed dormancy in dicot and monocot species. To further elucidate the regulation of seed dormancy in monocots, seed maturation regulators should be investigated in details.

## Supporting Information

Figure S1 Alignment of the deduced amino acid sequences of the B domains of *LEC1*, *L1L* and their orthologues. Characters filled with black and gray represent perfect matches and more than 50% matches, respectively, among genes.
(TIF)

Figure S2 Alignment of the deduced amino acid sequences of the B3 domains of *LEC2*, *FUS3*/*TaFUS3*, *ABI3*/*TaVP1*, and seven *LEC2* orthologues. Characters filled with black and gray represent perfect matches and more than 50% matches, respectively, among genes.
(TIF)

Table S1 Gene-specific primers used for quantitative RT-PCR.
(DOCX)

Table S2 Accession numbers of *LEC1*, *LEC2*, *FUS3*, *ABI3*/*VP1* and their orthologous genes.
(DOCX)
