# Supplementary figures and images for: Seed Maturation Regulators Are Related to the Control of Seed Dormancy in Wheat (Triticum aestivum L.)
Source: PLoS One. 2014 Sep 11;9(9):e107618. doi: 10.1371/journal.pone.0107618 (PMC4161473; doi:10.1371/journal.pone.0107618)

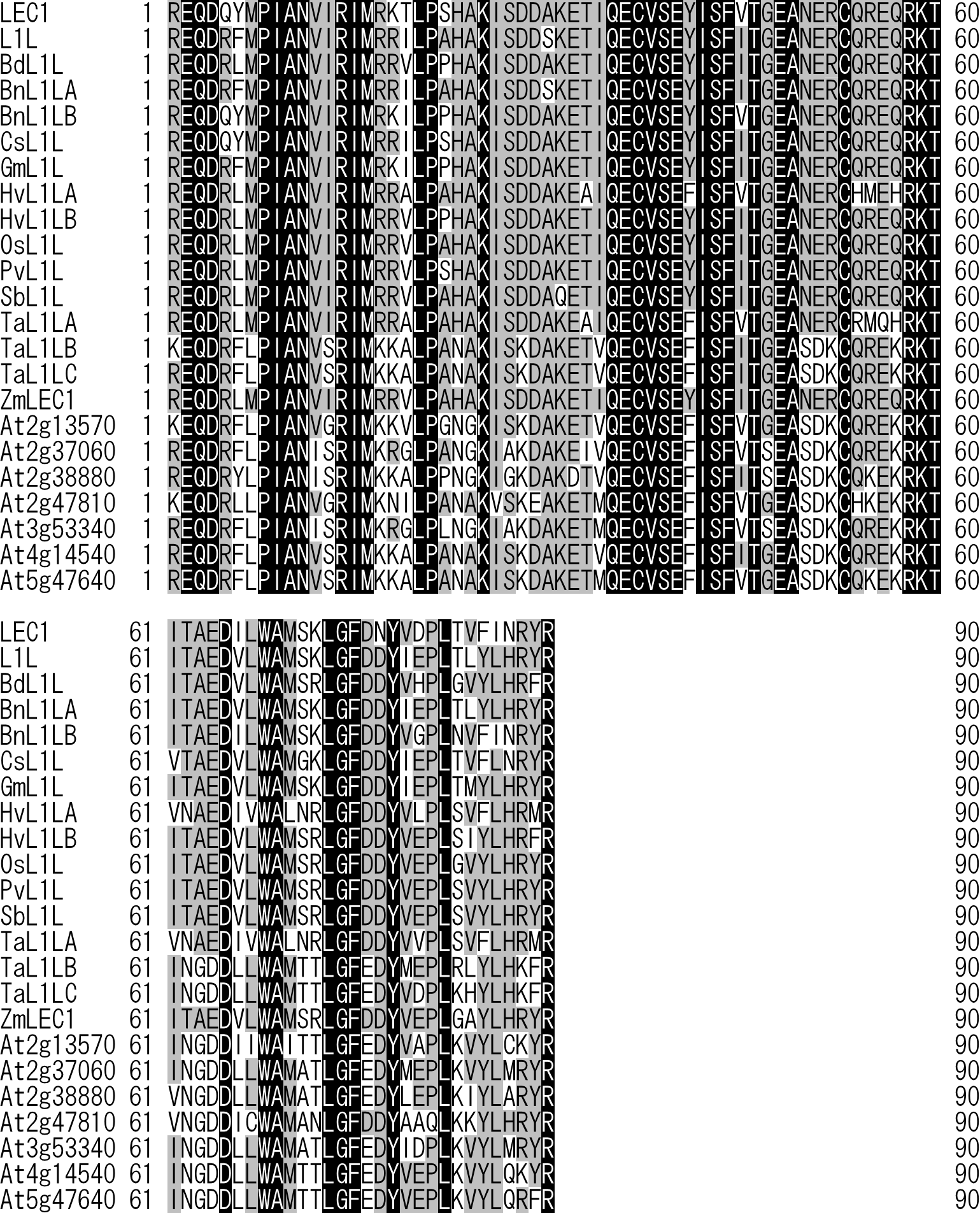

Supplement: Figure S1 — Alignment of the deduced amino acid sequences of the B domains of LEC1, L1L and their orthologues. Characters filled with black and gray represent perfect matches and more than 50% matches, respectively, among genes. (TIF) [file pone.0107618.s001.tif]

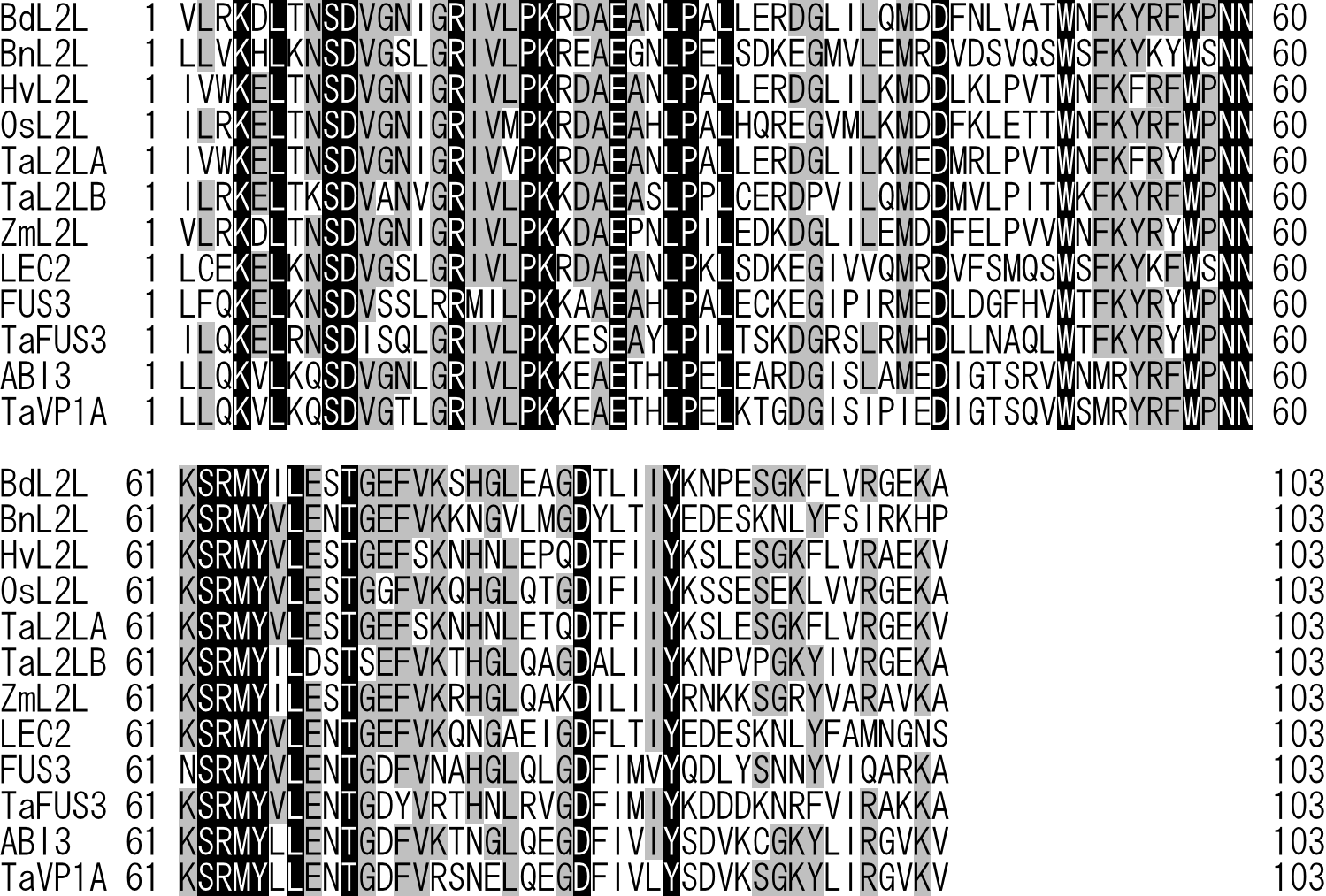

Supplement: Figure S2 — Alignment of the deduced amino acid sequences of the B3 domains of LEC2, FUS3/TaFUS3, ABI3/TaVP1, and seven LEC2 orthologues. Characters filled with black and gray represent perfect matches and more than 50% matches, respectively, among genes. (TIF) [file pone.0107618.s002.tif]
