# Supplementary material for: Seed Maturation Regulators Are Related to the Control of Seed Dormancy in Wheat (Triticum aestivum L.)
Source: PLoS One. 2014 Sep 11;9(9):e107618. doi: 10.1371/journal.pone.0107618 (PMC4161473; doi:10.1371/journal.pone.0107618)
Supplement: Table S1 — Gene-specific primers used for quantitative RT-PCR. (DOCX) [file pone.0107618.s003.docx]

Table S1 Gene specific primers used for quantitative RT-PCR

| Gene | Forward primer | Reverse primer |
| --- | --- | --- |
| *TaL1LA* | GCCAGCAGCAGAAGAATC G | CGCGACTAGCCAGATTGGA |
| *TaL2LA* | TTGTCCGGAATGCAAGGAG | TGAACCCGCATTGTTTGTTGT |
| *TaL2LB* | TACAACATCCCTGGCACCAA | CCTTCCATGGGAGGCATATG |
| *TaFUS3* | TCTTTGCCACAATTCCACGA | CTTCTGGAATTGGCAGAATGG |
| *TaVP1* | AATCCGCTGTCGACGAAGTC | CGAGGGTTTTTGCTGCTTCT |
| *TaDOG1* | CGGGCTCAAAACCATGACA | GCGGCAAAGCAAGCTAGTTG |
| *TaCDCP* | CAAATACGCCATCAGGGAGAACATC | CGCTGCCGAAACCACGAGAC |
