# Supplementary material for: Seed Maturation Regulators Are Related to the Control of Seed Dormancy in Wheat (Triticum aestivum L.)
Source: PLoS One. 2014 Sep 11;9(9):e107618. doi: 10.1371/journal.pone.0107618 (PMC4161473; doi:10.1371/journal.pone.0107618)
Supplement: Table S2 — Accession numbers of LEC1, LEC2, FUS3, ABI3/VP1 and their orthologous genes. (DOCX) [file pone.0107618.s004.docx]

Table S2 Accession numbers of *LEC1*, *LEC2*, *FUS3*, *VP1*/*ABI3* and their orthologous genes

| Name | Accession No. | Query coverage  (%) | E-value | Name | Accession No. | Query coverage  (%) | E-value |
| --- | --- | --- | --- | --- | --- | --- | --- |
| LEC1 type |  |  |  |  |  |  |  |
| *LEC1* | NM_102046 | - | - | *HvL1LA* | AL506199 | 38 | 5e-52 |
| *L1L* | AY138461 | 63 | 7e-63 | *HvL1LB* | BE603222 | 41 | 2e-58 |
| *BdL1L* | XM_003570425 | 39 | 4e-57 | *OsL1L* | NM_001054521 | 42 | 2e-58 |
| *BnL1LA* | FG575907 | 49 | 8e-64 | *PvL1L* | FE616493 | 43 | 5e-60 |
| *BnL1LB* | GU945398 | 100 | 8e-119 | *SbL1L* | XM_002452582 | 68 | 8e-64 |
| *CsL1L* | XM_004153666 | 58 | 3e-68 | *TaL1LA* | BT009029 | 57 | 3e-50 |
| *GmL1L* | NM_001249696 | 84 | 4e-70 | *ZmLEC1* | NM_001112048 | 49 | 2e-59 |
|  |  |  |  |  |  |  |  |
| non-LEC1 type | |  |  |  |  |  |  |
| At2g13570 | NM_126937 | 46 | 8e-45 | At4g14540 | NM_117534 | 40 | 2e-50 |
| At2g37060 | NM_201888 | 66 | 4e-50 | At5g47640 | NM_124138 | 40 | 2e-49 |
| At2g38880 | NM_001202776 | 45 | 2e-45 | *TaL1LB* | tplb0013d06 | 40 | 1e-47 |
| At2g47810 | NM_130348 | 46 | 1e-43 | *TaL1LC* | tplb0024f16 3093 | 41 | 4e-50 |
| At3g53340 | NM_115194 | 59 | 1e-49 |  |  |  |  |
| *LEC2* | NM_102595 | - | - | *OsL2L* | AK072874 | 43 | 6e-38 |
| *BdL2L* | XM_003579428 | 31 | 7e-39 | *TaL2LA* | JP236538 | 27 | 7e-34 |
| *BnL2L* | HM370539 | 99 | 9e-161 | *TaL2LB* | tplb0004123 | 47 | 3e-35 |
| *HvL2L* | PUT-169a-Hordeum_  vulgare-63956 | 33 | 1e-33 | *ZmL2L* | EU975282 | 40 | 3e-36 |
| *FUS3* | AF016265 | - | - | *OsLFL1* | AK109920 | 53 | 5e-44 |
| *BdFUS3* | Bradi2g48057 | 40 | 2e-46 | *SbFUS3* | XM_002456198 | 47 | 3e-40 |
| *BnFUS3* | HM370540 | 99 | 5e-168 | *TaFUS3* | JV941539 | 34 | 4e-46 |
| *HvFUS3* | AM418838 | 34 | 5e-46 | *ZmFUS3* | AY107817 | 51 | 1e-46 |
| *ABI3* | NM_113376 | - | - | *VP1* | NM_001112070 | 88 | 3e-105 |
| *PsABI3* | EU026375 | 90 | 1e-163 | *AfVP1* | AJ001140 | 63 | 1e-96 |
| *SlABI3* | AY911399 | 88 | 2e-117 | *HvVP1* | AK375797 | 66 | 4e-93 |
| *VvABI3* | XM_003632349 | 100 | 7e-168 | *OsVP1* | AK105441 | 64 | 5e-97 |
|  |  |  |  | *TaVP1* | AJ400712 | 74 | 2e-94 |

Additional abbreviation of each gene represents species name as follows; Af: *Avena faua*, Bd: *Brachypodium distachyon*, Bn: *Brassica napus*, Cs: *Cucumis sativus*, Gm: *Glycine max*, Hv: *Hordeum vulgare*, Os: *Oryza sativa*, Ps: *Pisum sativum*, Pv: *Panicum virgatum*, Sb: *Sorghum bicolor*, Sl: *Solanum lycopersicum*, Ta: *Triticum aestivum*, Vv: *Vitis vinifera*, Zm: *Zea mays*.
